# Ethical violations in the clinical setting: the hidden curriculum learning experience of Pakistani nurses

**DOI:** 10.1186/s12910-015-0011-2

**Published:** 2015-03-19

**Authors:** Sara Rizvi Jafree, Rubeena Zakar, Florian Fischer, Muhammad Zakria Zakar

**Affiliations:** 1 Institute of Social and Cultural Studies, University of the Punjab, P.O. Box 54590, Lahore, Pakistan; 2 Forman Christian College, Sociology Department, University of the Punjab, 21 FCC Maratib Ali Road, 54000 Gulberg, Lahore Pakistan; 3 School of Public Health, Department of Public Health Medicine, Bielefeld University, P.O. Box 100 131, 33501 Bielefeld, Germany

**Keywords:** Clinical setting, Ethics, Ethical violations, Hidden curriculum, Nurse

## Abstract

**Background:**

The importance of the hidden curriculum is recognised as a practical training ground for the absorption of medical ethics by healthcare professionals. Pakistan’s healthcare sector is hampered by the exclusion of ethics from medical and nursing education curricula and the absence of monitoring of ethical violations in the clinical setting. Nurses have significant knowledge of the hidden curriculum taught during clinical practice, due to long working hours in the clinic and front-line interaction with patients and other practitioners.

**Methods:**

The means of inquiry for this study was qualitative, with 20 interviews and four focus group discussions used to identify nurses’ clinical experiences of ethical violations. Content analysis was used to discover sub-categories of ethical violations, as perceived by nurses, within four pre-defined categories of nursing codes of ethics: 1) professional guidelines and integrity, 2) patient informed consent, 3) patient rights, and 4) co-worker coordination for competency, learning and patient safety.

**Results:**

Ten sub-categories of ethical violations were found: nursing students being used as adjunct staff, nurses having to face frequent violence in the hospital setting, patient reluctance to receive treatment from nurses, the near-absence of consent taken from patients for most non-surgical medical procedures, the absence of patient consent taking for receiving treatment from student nurses, the practice of patient discrimination on the basis of a patient’s socio-demographic status, nurses withdrawing treatment out of fear for their safety, a non-learning culture and, finally, blame-shifting and non-reportage of errors.

**Conclusion:**

Immediate and urgent attention is required to reduce ethical violations in the healthcare sector in Pakistan through collaborative efforts by the government, the healthcare sector, and ethics regulatory bodies. Also, changes in socio-cultural values in hospital organisation, public awareness of how to conveniently report ethical violations by practitioners and public perceptions of nurse identity are needed.

## Background

The clinical setting is recognised as being the place where the hidden curriculum is absorbed by medical practitioners during training and practice [1-3]. The hidden curriculum is the undocumented part of medical education which dictates professional practice and processes through strong and sustained socio-cultural forces [4-6]. Medical trainers, instructors and senior licenced practitioners influence the future ethical practices of both students and other work colleagues through the hidden curriculum [7-10]. Patient safety and optimal role delivery in the clinical setting is highly dependent on nurse training and professional ethics [11]. Nurses are front-line practitioners, known for spending the most time with patients and also being the main coordinating force for all other medical practitioners [12]. Consequently, nurses have substantial knowledge of the hidden curriculum and the practice of ethics in the clinical setting [13].

The issue of the hidden curriculum is highly relevant in Pakistani society due to strong socio-cultural forces that control healthcare organisational practices and medical ethics [14-16]. Pakistan is an underdeveloped nation, fraught with grave political, economic and regional difficulties [17]. The healthcare system is hampered by extremely low expense allocation (less than 2% of the government budget), unstructured planning and an absence of policies on many health-related issues, and corruption within the medical administration [18,19]. There is a paucity of literature in this field in Pakistan. The available literature indicates high levels of ethical violations in clinical practice [20]; including the absence of informed consent, a lack of patient rights, unprofessional guidelines, and the deliberate withdrawal of treatment [15,21-25]. Reasons for ethical violations in the Pakistani healthcare system have been discussed in terms of inadequate training of medical and nursing practitioners, long duty hours and low pay-scales, the absence of legal protection for patients, a lack of professional assessment of practitioners and the nonexistence of compulsory registration of medical and nursing practitioners [20,26,27].

Since public-sector hospitals are understaffed, medical and nursing practitioners are overburdened and as a resultant they put less emphasis on following structured care plans, reporting errors and observing medical ethics practice [16]. Like other sectors in the country, the healthcare sector is highly male-dominated; with patriarchal, paternalistic and conservative belief systems influencing working relations and output [26]. Because nursing is a feminised profession in Pakistan, it bears the brunt of patriarchal practices, with female nurses evidenced to be victimised, abused and professionally sidelined in the absence of laws and policies ensuring their protection and professional autonomy [28,29].

### Ethics in Pakistan’s healthcare sector

#### Regulatory bodies

The National Bioethics Committee (NBC) was established in 2004 by the government of Pakistan, with the aim of promoting ethics in the healthcare sector through two subcommittees: the Research Ethics Committee (REC) and the Medical Ethics Committee (MEC). The Pakistan Medical and Dental Council (PMDC) and the Pakistan Nursing Council (PNC) are, respectively, the statutory regulatory and registration authority for medical and nursing practitioners in the country. The PMDC and PNC are both independent of the government, and administered by medical practitioners. Medical and nursing curricula are also revised and supervised by the Higher Education Commission (HEC), a government body which licenses educational institutes and verifies degrees. Despite the presence of designated regulatory bodies for ethics promotion, the healthcare sector in Pakistan is hampered by the absence of ethics courses in the curriculum, the non-existence of hospital ethics committees or Institutional Review Boards (IRBs) in most hospitals, and the existence of only one non-indexed ethics journal (*Pakistan Journal of Medical Ethics*) [15,30].

### Ethics education

To qualify as a registered doctor or surgeon in Pakistan, medical students need to complete five years of a bachelor’s degree in medicine and surgery (referred to as an MBBS) and one year of clinical training (referred to as a ‘House Job’). Registered nurses must have either a two-year Nursing Diploma or a three-year bachelor’s degree in Nursing (BSc Nursing). Although the PMDC and PNC have made ethics education compulsory, the majority of medical and nursing institutes in the country do not teach compulsory courses in ethics or conduct formal examinations on ethics [27,31,32]. In addition, there is no monitoring or assessment of ethical compliance during clinical training and practice. In developing countries, including Pakistan, where there is weak regulatory infrastructure and curriculum exclusion, medical practitioners usually rely on international ethical documents as broad guidelines for ethical awareness [27,33].

### Objective of this study

Given that ethics training is only superficially covered in the formal medical and nursing curricula, and that the regulation of ethics is also weak in the country, research about how the hidden curriculum may be teaching ethical violations became an important topic for us to pursue. To the best of this researcher’s knowledge, nurses’ perceptions and experiences of ethical violations in the region have not been researched. There is a danger that if the ethical violations that are taught through the hidden curriculum remain neglected by researchers, this will reinforce cyclical violations in the future [5]. In addition, nurses’ experiences of ethical violations in the clinical setting need to be identified before they can be resolved [34,35]. Vaughn’s structural secrecy theory has been used in nursing research to propose that each region must separately ascertain its native layers of the hidden curriculum, which influences practitioner position and output [36,37]. The aim of this study was to identify those aspects of the hidden curriculum which encourage ethical violations in the clinical setting, through the ‘life-world’ experiences of nurses, during clinical training and practice.

## Methods

### Study

This study is part of a doctoral dissertation entitled “Nurses’ perceptions of organisational culture and its association with error reporting: A study of tertiary-care public sector hospitals in Lahore”, written by the first author. Since there was an absence of literature on nurses’ perceptions of medical negligence in Pakistan, a qualitative phenomenological approach was considered appropriate to capture the organisation-specific and culturally relevant experiences of nurses [38]. Additionally, within empirical research in clinical ethics, there is a growing recognition that qualitative methods are beneficial in identifying the socio-cultural forces driving the hidden curriculum [39,40]. Ethics committee permission was obtained from the hospitals and nursing institutes where data collection took place, and also from the Institutional Review Board, University of the Punjab.

### Setting

The study was conducted in two prominent tertiary-care public-sector hospitals in Lahore, both of which have affiliated medical and nursing schools. The hospitals were randomly sampled from a list provided by the Pakistan Institute of Medical Sciences, which shows a total of nine tertiary-care public-sector hospitals listed for Lahore. The hospitals will be referred to as Hospital A and Hospital B, to preserve the anonymity of the participants. Both hospitals cater to a large number of patients from both the rural and urban Lahore District and also from the surrounding villages of Lahore City. Combined, the two hospitals have a daily out-patient turnover rate of 3,800 patients and an in-patient capacity of 1,890 beds.

### Sample

The sampling inclusion criterion was all willing female registered nurses and registered nurse students who had been working in the clinical setting for more than one year. Nurses from all five designations were included, i.e.: nurse supervisor, nurse instructor, nurse ward head, staff nurse and student nurse. Both hospitals combined have a total of two nurse supervisors, 33 nurse instructors, 250 nurse ward heads, 1,250 staff nurses and 735 student nurses. Nurse supervisors and nurse instructors were asked for interviews personally by the first author. Both nurse supervisors were asked and they showed an interest in participating in the study. A total of 14 nurse instructors were approached for interview, but only nine showed any interest and finally eight participated in the study. Informed consent was obtained from all respondents. Nurse ward heads, staff nurses and student nurses were invited for interviews through notices placed on bulletin boards in the nursing school corridors, nursing school libraries, and in the offices of nurse ward heads (which all nurses have to visit daily to sign attendance registers). Notices were displayed on boards for a period of five weeks. Additionally, nurse ward heads were requested by the first author to encourage staff nurses and student nurses to participate in the study. All the nurses who responded to the notices and demonstrated a willingness to participate in the study, by texting the first author, were interviewed. Finally, a total of 42 participants were sampled, consisting of two nurse supervisors, eight nurse instructors, ten nurse ward heads, 11 staff nurses and 11 student nurses. Twenty of the nurse participants had a Nursing Diploma, 19 had a BSc in Nursing and three had an MSc in Nursing.

### Interviews

The research question for this study was designed to discover whether any ethical violations are taught through the hidden curriculum, as experienced by nurses during clinical training and practice. Although this question was phrased in a ‘leading’ manner, it was considered important to do so, as collecting data on sensitive topics in conservative and male-dominated societies does not invite open discussion, due to fears of retribution and job loss [41,42]. The PNC code of ethics [43], the ICN code of ethics [44], and the UNESCO core values of medical ethics [45] were consulted and summarised to distribute to participants in semi-structured interviews (Appendix A). Participants were asked what kind of ethical violations, if any, they might have experienced during clinical practice, with specific regard to: 1) professional guidelines and integrity, 2) patient informed consent, 3) patient rights, and 4) co-worker coordination for competency, learning and patient safety.

Twenty interviews and four focus group discussions (FGDs), with 5–6 members each, were conducted (Table 1). Confidentiality and anonymity issues were discussed with all participants before the start of the interviews and FGDs. The participants were assured that they could leave the discussion at any point during the proceedings. Discussions lasted between 35 and 65 minutes. All interviews were carried out in the English language, as English is both the official working language and the academic language for medical and nursing students in Pakistan. In general, audio recording was not used, except when the nurses allowed it (in some FGDs), because nurses were wary of recording devices, given the confidential and professionally sensitive topic of discussion. Due to seniority and time constraints, nurses belonging to the senior designations of nurse supervisor, nurse instructor and nurse ward head were interviewed according to their convenience in face-to-face private individual interviews. Staff nurses and student nurses were interviewed in FGDs. All interviews took place over a period of four weeks in private rooms in the nursing schools, located at a distance from the hospital setting, which allowed confidentiality and privacy from the busy and public clinical setting.

**Table 1:** **Focus group discussions and interviews**

		Nurse supervisors	Nurse instructors	Head nurses	Staff nurses	Student nurses	Total participants
Hospital A	FGD (2)				6	6	12
	Interviews	1	5	6			12
Hospital B	FGD (2)				5	5	10
	Interviews	1	3	4			8
Total participants		2	8	10	11	11	42

### Data analysis

A deductive qualitative research design using content analysis was used due to the presence of an organised framework of categories for the nursing code of ethics [46,47]. Four broad nursing codes of ethics were used as preliminary categories to guide and prompt the participants to discuss issues specific to the study aim. Content analysis is commonly used in nursing research and has the benefit of flexibility to suit different research designs [48]. It has commonly been used to understand and identify meanings in communication and the processes of the hidden curriculum in the health sector [5,49,50].

The research process consisted of the following steps: the complete interviews were taken as the unit of analysis, all interview notes and audio interviews were transcribed, the text was read repeatedly to identify categories of relevance and importance, and the coding of sub-categories was developed under the four predefined categories of the nursing code of ethics [51,52]. Categories and coding were confirmed in context to identify hidden meanings and to consider nurses’ experiences [53]. An example of how sub-categories were developed and coded is described in Table 2. Reliability checks were conducted by seeking clarification during interviews and paying attention to details. Note transcripts were repeatedly analysed to identify significant categories of relevance. The reliability of findings and final drafts of categories was assured through multiple coding by the first researcher, researcher assistants and senior researchers, and finally through respondent validation [51,54].

**Table 2:** **Example of coding data into sub-categories**

Do you regularly take informed consent from patients?	Consent taking	Absence of consent-taking
	Only during surgeries, in writing	Absence of consent-taking in writing, except for surgical procedures
	Not for emergencies, unless surgery required	
	Not for out-patient medical administration	
	Not for in-patient medical administration and all other non-surgical procedures	

## Results

From the four predefined categories in the nursing code of ethics, a total of ten strong and clear sub-categories were found (Table 3). All participants had knowledge of the nursing code of ethics and there was general agreement about the ethical violations taught through the hidden curriculum in the clinical setting. Under the category of ‘*professional guidelines and integrity*’, it was found that student nurses were used as adjunct staff, nurses had to face frequent violence in the hospital setting and patients were reluctant to receive treatment from nurses. The category of ‘*patient informed consent*’ revealed that there was a near-absence of consent taken from patients for most non-surgical medical procedures and an absence of patient consent taking for receiving treatment from student nurses. Under the category of ‘*patient rights*’, it was found that patient discrimination was practised on the basis of a patient’s socio-demographic status (literacy and socio-economic status) and that nurses practised withdrawal of treatment out of fear for their safety. Lastly, under the category of ‘*co-worker coordination for competency, learning and patient safety*’, it was found that nurses experienced a non-learning culture, blame-shifting from seniors and that they practised non-reportage of errors.

**Table 3:** **Categories and sub-categories**

Categories	Professional guidelines and integrity	Patient informed consent	Patient rights	Coworker coordination for competency, learning and patient safety
**Sub-categories**	Nurse students used as adjunct staff	Near-absence of consent taking from patients for most non-surgical medical procedures	Patient discrimination on the basis of socio-demographic status	Non-learning culture
	Nurses having to face violence	Absence of patient consent taking for receiving treatment from student nurse	Withdrawal of treatment by nurses who fear for their safety	Blame shifting and non-reportage of errors
	Patient reluctance to receive treatment from nurse			

### Professional guidelines and integrity

#### Student nurses used as adjunct staff

All participants described how student nurses were commonly used as adjunct staff in the hospital setting. Participants stressed that student nurses were not trained or experienced enough for this, and that staff duties restricted students from having the time to study and observe during clinical training. A third-year student nurse described the situation thus:

We hardly have time to read our course books or study for exams, there is so much clinical work pressure. Apart from clinical duty, we are even assigned jobs like recording pharmacy expenses during off-duty clinical hours.

### Nurses having to face violence

Nurse participants concurred that there was frequent use of verbal and physical violence against female nurses by male medical doctors and physicians, male patients and male family attendants. Nurses talked freely of being slapped or physically beaten, not just by patients from the neurology department, but by educated and mentally stable patients and family attendants. It was agreed that all patients were accompanied by multiple family attendants. A staff nurse described an episode of a senior nurse being physically manhandled by a male attendant:

A few days ago, a senior nurse was passing an IV line when a male attendant burst into the emergency room with his mother. The attendant’s mother was in extreme pain. The attendant physically pulled the nurse, disrupting the IV she was administering. The nurse assured the attendant that his mother was stable and not in need of urgent medical attention. Despite this, the attendant forcefully held the nurse from going back to her first patient until she gave his mother a painkiller and a sedative. Only after his mother fell asleep did the attendant let go of the nurse.

An experienced participant gave her opinion about the reason behind male aggression against female nurses:

Men in our society are used to beating up and abusing their women relatives at home. Female nurses are treated like women relatives, or, even worse, are likened to house-maids. As nurses we are expected to obey orders, clean and wash and most importantly to accept physical and verbal abuse without protest.

### Patient reluctance to receive treatment from nurses

A negative nurse identity was described by participants as being widely prevalent in the region, causing reluctance in patients to receive treatment from nurses. Participants revealed that patients do not trust nurses to do their job in the same way as they trust doctors and surgeons. A staff nurse described an incident:

A nurse colleague was about to administer a painkiller injection, when the patient abused her and said: “Don’t you know this causes muscle pain for seven days?” The patient demanded to be given an alternative medicine. Although the staff nurse attempted to explain that the alternative medicine had harmful side-effects, the patient was unwilling to listen. The nurse had to wait until the doctor discussed ongoing treatment with the patient and as a result medicine was not administered at the prescribed time.

### Patient informed consent

#### Near-absence of consent taking from patients for most non-surgical medical procedures

Nurse participants described how patient consent was only taken in writing for surgical procedures. All non-surgical procedures, medical administration, out-patient services and emergency care were administered without informed consent in writing or without a discussion with patients. It was reasoned by a nurse participant that:

It is not possible to get written approvals or discuss treatment options, due to shortage of staff, lack of time and the problems of dealing with a majority of poor and illiterate populations.

### Absence of patient consent taking for receiving treatment from student nurses

It was confirmed by all participants that patient consent was never taken for the administration of treatment by student nurses. Discussions revealed the belief that, since the patient had voluntarily come to a teaching hospital, indirect consent had been given. A discussion group of nursing students elucidated that:

In the first year of clinical training, nurse students should not administer injections, but our instructors expect us to do it; and mostly without their supervision, due to shortages. Patients are unaware that we are nurse students.

### Patient rights

#### Patient discrimination on the basis of socio-demographic status

Participants stated that care delivery in the hospital was dictated on the basis of a patient’s socio-demographic status, with the rich and privileged receiving better care, staffing and resources. Patients who were poor, illiterate and of lower socio-economic status were allocated student nurses for the administration of treatment; whereas patients who were literate and of higher socio-economic status were given more care by the hospital administration and senior doctors. A nurse supervisor confirmed that:

Politicians, government employees or relatives of doctors and hospital administrators receive preference in hospital care. They don’t have to wait long hours and the best resources and private rooms are reserved for them.

### Withdrawal of treatment by nurses who feared for their safety

Participants described how their experiences of patients and attendants becoming physically and verbally aggressive had forced the senior nurses to develop in themselves, and encourage in junior nurses, a professional bearing of aloofness and distance during role delivery. Clinical rounds by nurse participants were described as striving for minimal interaction with patients and family attendants due to the fear of violence. An example was given by a group discussion member of the abandonment by nurses of open communication and emotional support for family attendants, due to the fear of facing violence:

Last week a patient had expired, but the family attendants were so aggressive and explosive that the nurses on duty continued resuscitation and had the patient shifted to an empty surgical ward. It was communicated to the family attendants that the patient was critical. Meanwhile the nurse ward-head called in male medical students to disclose news of the demise of the patient to family attendants.

Another incident was described in which a senior nurse pretended to be administering medical treatment to placate a volatile patient:

A TB patient became verbally abusive because the nurse ward head was not giving him pain relief before the prescribed 6-hour wait. Thus the ward head gave him water in an injection and, psychologically satisfied, he went to sleep.

### Co-worker coordination for competency, learning and patient safety

#### Non-learning culture

An experience common to nurse participants from both institutes was that the nurse trainers (including nurse instructors, nurse supervisors, doctors and surgeons) conducted a non-learning method of clinical training that did not permit questioning. Trainees and student nurses who questioned trainers were quickly shamed and put in their place. One participant reminisced about her experience of questioning a trainer during her first year of study:

I was very rudely reprimanded for asking an important question during one of my preliminary classes. The trainer asked me if I was in the habit of asking silly questions and why I had not been pre-reading my course-books. I felt humiliated and made sure that I did not ask questions again in the coming years of study.

Participants described how the non-learning culture contributed to inadequate knowledge and forced ethical violations, which weighed heavily on the personal ethics of nurses. A staff nurse recounted how she continued to feel guilty after administering the wrong medication:

The nurse ward-head read the prescription chart to me and moved on to the next case. I was unsure about need for dilution, but was too scared and embarrassed to ask. I administered the KCl (potassium chloride) injection without 100 ml dilution. Thankfully, no adverse consequence to patient occurred, but it was a horrible experience which gave me sleepless nights.

### Blame shifting and non-reportage of errors

Participants portrayed the job description of nurses as including taking the blame for errors made by doctors and surgeons. It was asserted that doctors and surgeons managed to successfully blame-shift because they were backed by their seniors, the medical administration and a strong union; whereas nurses did not have similar support networks. The consequences of sharing or reporting errors included character defamation and workplace network stigmatisation. The nurses shared a well-known incident of a junior nurse becoming the recipient of wrongful slander and having to leave the profession because she had violated the invisible code of reporting a breach of conduct by a senior doctor:

A senior doctor left the ward after an altercation with an attendant, without diagnosing treatment for a critical patient, knowing there was no replacement doctor. The nurse reported what she felt was a breach of ethical conduct to the medical superintendent. In return, the doctor had her banned from his ward. Additionally, the doctor had informal gossip circulated in terms of that particular nurse having a habit of regularly questioning doctors’ orders because she was a middle-aged frustrated spinster who had no other source of excitement in her life. Afterwards the nurse was not welcome in other ward teams and as her professional life became difficult in the organisation, with little chance of government transfer to another hospital, she left the profession altogether.

## Discussion

Our study found that, although medical ethics is not being formally taught, examined or monitored for compliance, nurses were aware of what constitutes ethical practice and were also knowledgeable about ethical violations taught through the hidden curriculum. Practising nurses from all designations (supervisor, instructor, ward head, staff and student) and from three different degree backgrounds (Diploma in Nursing, BSc Nursing and MSc Nursing) were represented. Responses across designations were satisfactorily similar. The findings also revealed that serious ethical violations exist in the clinical setting, possibly because of regressive cultural norms and social identities, and inadequate health governance [16,20,27].

**Professional guidelines and integrity** were compromised through the use of student nurses as adjunct staff. Nursing students’ involvement in such jobs may have consequences for their training as they are unable to adequately train during their student years due to the responsibilities of staff duties, and this also forces error-making in the workplace due to inadequate knowledge [55]. Other literature from South Asian countries confirms that, although clinical training is part of the curriculum of nursing students, due to shortages, nurses are made to perform the duties of staff nurses without supervision [56-58].

Although, ideally, medical policies and local laws aim to guarantee nurse safety and integrity in the clinical setting, our findings reveal that nurses reported experiencing high levels of verbal and physical violence during role delivery. Cultural tendencies towards violence against women, especially in patriarchal societies, tend to cross over into professional relations, especially in nursing [22,29]. Nurses also experience violence from patients and family members due to shortages of staffing, being overburdened in their duties and the practice of reserving better services for patients of elite socio-economic status [59]. Nurses in Pakistan have to manage violence from three sources: co-workers and trainers, patients, and multiple family attendants. Social acceptance of the low status of nurses and fears of violence from multiple sources leads to nurses adopting coping strategies such as non-disclosure and withdrawal of treatment from patients [60,61].

Our findings reveal that patients are reluctant or unwilling to receive treatment from nurses, due to the inferior status of nurses and a negative nurse identity in the community. The reluctance of patients to receive treatment from nurses is known to have a negative impact on patient safety due to delayed treatment in the absence of a doctor, and even to cause patient mortality [62]. Previous research confirms that patients from developing and patriarchal regions prefer medical administration to be performed by doctors, while preferring nurse duties to be reserved for tasks like body-sponging and bed-linen changing [63]. Reasons for patient reluctance to receive treatment from nurses includes the general belief that nurses are medically incompetent [64], a lack of nurse training in dealing with different languages, customs and sectarian beliefs [65], and patients witnessing the general attitude of doctors in treating nurses as inferior colleagues [66].

**Patient informed consent** is being seriously violated, according to our study’s findings. All participants confirmed that there is a near-absence of consent taking from patients for most non-surgical medical procedures and an absence of patient consent taking for receiving treatment from student nurses. Discussions revealed that this was mainly due to time constraints, and the difficulties of having to communicate with populations that are largely illiterate. In Asian societies, there is a lot of pressure to take informed consent from multiple family members, due to ‘family autonomy’ taking precedence over ‘individual autonomy’, and thus medical practitioners prefer to take swift decisions autonomously [25,33]. However, research shows that the lack of consent taking or discussion of treatment options with patients contributes in the long run to patient hostility, distrust and feelings of lack of control [67]. In addition, the absence of consent taking also weighs heavily on nurse practitioners’ professional ethics and becomes a burden on them psychologically; consequently, this influences their commitment to work [68].

**Patient rights** were found to be breached through the withdrawal of treatment. Firstly, hospital service delivery and the allocation of staffing and resources were being distributed according to a patient’s socio-demographic characteristics. The poor, the illiterate and populations of lower socio-economic status, who usually visited public-sector hospitals, were being deprived of optimal care provision, in comparison to richer populations and those of upper socio-economic status. This is consistent with previous research, which found that confounding problems of staffing and resource shortages, role burden, difficulties in dealing with illiterate patients, and pressure from VIP society (which control promotion and the retention of medical practitioners) contribute to the practice of patient discrimination in public health services [69]. Secondly, nurses were practising withdrawal of treatment from patients out of concern for their own safety and fears of facing violence. As a consequence, nurse communication, cultural competency, emotional relief and care provision for patients were limited. The absence of care provision and inadequate role delivery by the nurse practitioner is of grave concern due to the negative consequences on patient safety, and also on the job satisfaction and job commitment of the nursing professional [70].

**Co-worker coordination for competency, learning and patient safety** is an integral component of nursing ethics. Clinical trainers are instrumental in teaching ethics and creating a culture of learning and sharing; which consequently promotes ethical compliance and error reporting [71]. Our findings, however, show that nurses are training in a non-learning and hierarchical culture, where knowledge-sharing and competency development is dangerously limited. Barriers to learning and sharing are created through informal social laws that sanction outspoken and enquiring juniors through character defamation. Other studies have highlighted that non-learning cultures are common in hospital settings when there is a shortage of staff, a heavy workload and hierarchical cultures [72].

Our findings also suggest that doctors and surgeons shift blame onto nurses as a norm, specifically when nurses attempt to report or share errors in the workplace. Fears of losing their job or lack of promotional opportunities create disincentives amongst nurses to report errors. Character defamation for female members of Pakistani society is not a minor problem, as it causes family dishonour, social ostracism, and lack of arranged marriage prospects, and fear of these major consequences limits nurse resistance to the status quo [73]. Other research has also evidenced that whistle-blowing in the hospital setting is frowned upon and ethical violations are underreported due to the pressures of a hierarchical culture and the fear of dismissal [74]. Pakistan’s healthcare sector suffers from an absence of formal systems for tracking and reporting errors and also from an absence of the culture of error reporting and error sharing; both of which are harmful to patient safety and optimal healthcare coordination and planning [75].

The limitations of this study include the fact that the findings are based on nurses’ reports of their experiences of clinical training and not on direct observation. This study is cross-sectional in nature and provides information on the existing situation. Other limitations include the absence of a larger sample across other public and private hospitals in Pakistan, which would be necessary to confirm the findings of our study and, lastly, as is the case with most qualitative research, researcher bias may be involved in category development. We tried to decrease this bias by sharing the categories and sub-categories found within the data with the available participants.

### Recommendations

#### State governance

The Ministry of Health and provincial governments must extract increased government budget allocations for the health sector. Critical areas requiring an increase in expenditure include: staffing, resources, education, and ethics regulation [69]. There needs to be a greater budget allocation and accountability for the actions and policy enforcement of ineffective bodies like the REC and MEC. Violence against nurses and other working women needs to be dealt with through a structured legal constitution. Laws have not been passed against the workplace harassment of women and thus collaborative bodies like the Ministry of Women, PNC and other women’s development agencies need to apply pressure for parliamentary bills to be passed and enforced in practice [76,77].

### Healthcare governance

Important stakeholders, such as medical educationalists, ethicists, HEC, REC, MEC, PMDC, and PNC, must enforce the establishment of ethical committees in all hospitals to monitor clinical ethics. PMDC and PNC must be given a mandate to deal with violating practitioners in a punitive manner, with the use of sanctions such as suspension or the complete revoking of licences. MEC must be given a mandate to revoke hospital licences if staff evaluation is not linked to the practice of clinical ethics. REC needs to encourage and fund the publication of more accredited ethics journals in the region, with international peer review, to encourage greater awareness of ethical violations in practice and more research in this area.

Family attendants on hospital premises need to be strictly limited and allocated scheduled visiting times by hospital administrations. Visiting regulations must be managed in a culturally sensitive manner, through raising awareness of the dangers of infection, possible harm to patients in causing staff diversions, and the assurance that female staff are always available for female patients [78,79].

### Curriculum development

Compulsory inclusion of medical ethics training in the syllabus must be enforced across all medical and nursing institutes, across all years of study, by HEC. Curriculum neglect should be penalised by HEC, through revoking the licences of educational institutions. Ethics examinations for students (for example: written, oral, ethics rounds, short papers, and case presentations) and for practising clinicians through continued education (for example: lectures, discussion groups, role play, standardised patients, internet-based cases, ethics rounds, and case study discussions) must be made mandatory [9,80,81]. Although some argue that the examination of ethics has little import, it is generally considered to be advantageous because it assesses the ability to practise ethics in the clinical setting under pressure [82], and also ensures that efforts are made to acquire ethical competency by both students and practitioners [27].

In addition, we recommend that the curriculum of medical ethics should be tailored to the social and cultural background where it is taught [83,84]. The already-established code of ethics from the developed world must be replaced by Islamic bioethics and the ‘Oath of the Muslim Doctor’. For example, religious script, such as ‘to save one life, is to save all of humanity’ would be influential in encouraging practical compliance by Muslim practitioners who need cultural and religious affirmation [85]. Palliative care and cultural competency training are non-existent in the Pakistani curriculum. The inclusion of training in palliative care for nurses and other medical practitioners would reduce problems of the withdrawal of treatment and the pretence that practitioners are providing a cure when they are not [24]. Training in cultural competency is important for a country like Pakistan, which has complex ethnic and provincial diversity, and in the long run this would also improve patient trust in nursing competency and their willingness to receive treatment [86].

### Organisational culture

The organisational culture of public-sector hospitals must be transformed to encourage the hidden curriculum to teach ethical compliance, through open communication, knowledge sharing and a culture of error reporting [87-90]. The nurse-doctor relationship can be altered to ensure respect, positive teamwork and patient-safety collaboration, through regular workshops, conferences and meetings arranged by PMDC, PNC, individual hospital administrations, and even pharmaceutical companies. Public-sector hospitals need to retain clinical trainers who have formal ethics training, promote them as role models and create an organisational culture of praising and promoting them based on ethics successes [39,91]. Trainers and students should be coached together about the importance of the hidden curriculum in teaching ethical compliance, and also to encourage mutual awareness, respect and collaboration [92]. Anonymous feedback by students for the monitoring of trainers is also recommended, and this should be supervised by PMDC and PNC and also by MEC [93].

### Public awareness and nurse identity

Patients and family attendants would less frequently resort to violence if they were more aware of what constitutes ethical violations and how to report them in a convenient manner. User-friendly reporting channels and confidence in zero tolerance for ethical violations, with expedited action against offending practitioners and administration, must be made available at all public-sector hospitals [94]. This must be funded and supervised by NBC and also monitored by PMDC and PNC.

There is an urgent need to change the negative identity of nurses and to challenge patient reluctance to receive treatment from nurses. This can be achieved through counselling support for family attendants and nurses around issues like collaborative decision-making, role expectations, role limitations, awareness of nurse competency and mutual respect [95]. Public awareness of the importance of respect for working women and the status of nurses can be raised through reinforcement and awareness by learned religious scholars of Islam [96] and through communication of the honour that Islam bestows upon female nursing practitioners and care providers [97]. Finally, the media, women’s development agents, civilian groups and NGOs must help to improve nurses’ social identity through documentaries and awareness campaigns [98].

## Conclusion

Ethical violations taught through the hidden curriculum of Pakistan’s healthcare system, as perceived by nurses, have not previously been studied. This study has made a meaningful contribution by helping to identify nurses’ knowledge of the ethical violations being taught in the clinical setting. However, reforms in government policies, healthcare policies and curriculum development are more easily achieved; it is the community norms and organizational attitudes which are more difficult to change. Critical socio-cultural improvements are called for in terms of the social identity of nurses, practitioner commitment to patient consent and patient safety, and trainer and co-worker collaboration in promoting a culture of learning and error sharing.

## Appendix A

Background information provided to participants about the international nursing code of ethics

The internationally agreed and endorsed nursing code of ethics includes: 1) professional guidelines and integrity, 2) patient informed consent, 3) patient rights, and 4) co-worker coordination for competency, learning and patient safety. ‘Professional guidelines and integrity’ refers to nurses following lawful professional guidelines for the safety of the patient and also for optimal role delivery of the nursing professional. The nurse respects and maintains her own dignity and that of the patient through concern for self-determination and autonomy. ‘Patient informed consent’ refers to the nurse taking informed consent from patients for any and all service delivery and making an attempt to keep the patient aware of the medical procedures being undertaken. ‘Patient rights’ refers to nurse respect for all patients and equal treatment without discrimination based on age, gender, socio-economic status, family status, disability or literacy. ‘Co-worker coordination for competency, learning and patient safety’ refers to adequate interrelations in the hospital setting amongst healthcare co-workers in an effort to maximise training and knowledge and also sharing errors to guarantee optimal role delivery and patient safety practice.

## Abbreviations

FGD: Focus group discussion
HEC: Higher Education Council
ICN: International Council of Nurses
MEC: Medical Ethics Committee
NBC: National Bioethics Committee
PMDC: Pakistan Medical and Dental Council
PNC: Pakistan Nursing Council
REC: Research Ethics Committee
UNESCO: United Nations Education, Scientific and Cultural Organization
